# *Staphylococcus aureus* Cas9 is a multiple-turnover enzyme

**DOI:** 10.1261/rna.067355.118

**Published:** 2019-01

**Authors:** Paul Yourik, Ryan T. Fuchs, Megumu Mabuchi, Jennifer L. Curcuru, G. Brett Robb

**Affiliations:** RNA and Genome Editing, New England Biolabs Inc., Ipswich, Massachusetts 01938, USA

**Keywords:** CRISPR, Cas9, *S. aureus*, *S. pyogenes*, gene editing, sgRNA

## Abstract

Cas9 nuclease is the key effector of type II CRISPR adaptive immune systems found in bacteria. The nuclease can be programmed by a single guide RNA (sgRNA) to cleave DNA in a sequence-specific manner. This property has led to its widespread adoption as a genome editing tool in research laboratories and holds great promise for biotechnological and therapeutic applications. The general mechanistic features of catalysis by Cas9 homologs are comparable; however, a high degree of diversity exists among the protein sequences, which may result in subtle mechanistic differences. *S. aureus* (SauCas9) and especially *S. pyogenes* (SpyCas9) are among the best-characterized Cas9 proteins and share ∼17% sequence identity. A notable feature of SpyCas9 is an extremely slow rate of reaction turnover, which is thought to limit the amount of substrate DNA cleavage. Using in vitro biochemistry and enzyme kinetics, we directly compare SpyCas9 and SauCas9 activities. Here, we report that in contrast to SpyCas9, SauCas9 is a multiple-turnover enzyme, which to our knowledge is the first report of such activity in a Cas9 homolog. We also show that DNA cleaved with SauCas9 does not undergo any detectable single-stranded degradation after the initial double-stranded break observed previously with SpyCas9, thus providing new insights and considerations for future design of CRISPR/Cas9-based applications.

## INTRODUCTION

Clustered regularly interspaced short palindromic repeats (CRISPR) and CRISPR-associated proteins (Cas) constitute sequence-based adaptive immunity in bacteria and archaea. Our understanding of CRISPR/Cas systems is progressing rapidly, in part driven by the excitement about adapting them for use in research, biotechnology, and human therapy (Wang et al. 2016; Chen and Doudna 2017; Garcia-Doval and Jinek 2017; Karvelis et al. 2017; Koonin et al. 2017; Shmakov et al. 2017; Hille et al. 2018; Mir et al. 2018). Of the diverse families of Cas nucleases, *Streptococcus pyogenes* Cas9 (SpyCas9) is the best-characterized and most widely used. SpyCas9 is a monomeric protein that can be programmed with a single guide RNA (sgRNA) to induce sequence-specific double-stranded (ds) breaks in DNA (Jinek et al. 2012). *Staphylococcus aureus* Cas9 (SauCas9) is a less well-characterized homolog. The proteins share 17% sequence identity as well as structural and mechanistic parallels (Nishimasu et al. 2015; Ran et al. 2015).

High-resolution structures and biochemical studies demonstrated that both SpyCas9 and SauCas9 bind sgRNA by interacting with the 3′-stem–loops, which induces a conformational change in the protein (Jinek et al. 2014; Jiang et al. 2015; Nishimasu et al. 2015; Mekler et al. 2016). The Cas9-sgRNA ribonucleoprotein (RNP) complex rapidly screens DNA in search of the protospacer adjacent motif (PAM). Following PAM recognition, the RNP attempts to base-pair the sgRNA's ∼20-nt 5′-terminal targeting sequence with the DNA in a 3′- to 5′-direction with respect to the sgRNA. If the DNA sequence adjacent to the PAM is complementary to the sgRNA an RNA:DNA duplex is formed—displacing one of the DNA strands—resulting in an R-loop (Gasiunas et al. 2012; Szczelkun et al. 2014; Sternberg et al. 2014; Jiang and Doudna 2017; Zeng et al. 2017; Gong et al. 2018). SpyCas9 recognizes a 5′-NGG PAM (Mojica et al. 2009; Anders et al. 2014; Sternberg et al. 2014) motif, while SauCas9 recognizes 5′-NNGRRT (Friedland et al. 2015; Nishimasu et al. 2015; Xie et al. 2018). Successful R-loop formation—contingent on perfect (or near-perfect) sgRNA:target DNA match—facilitates DNA cleavage marked by the RuvC domain and HNH domains cleaving the PAM-containing and the non-PAM-containing DNA strands, respectively (Gasiunas et al. 2012; Nishimasu et al. 2014; Sternberg et al. 2014). Work in vivo and in vitro, including single molecule and bulk kinetic experiments, showed that upon DNA cleavage SpyCas9 remains bound to the DNA, resulting in extremely slow product release, which ultimately inhibits enzymatic turnover (Sternberg et al. 2014; Richardson et al. 2016; Jones et al. 2017; Gong et al. 2018; Raper et al. 2018). Furthermore, recent work demonstrates that SpyCas9 modestly degrades cleaved DNA products (Jinek et al. 2012; Ma et al. 2015; Stephenson et al. 2018).

Here, using biochemistry and enzyme kinetics, we compared the DNA cleavage activity of SpyCas9 and SauCas9 RNPs, in vitro, on a 110-nt-long dsDNA containing a PAM sequence that is recognized by both homologs. Our data suggest that both homologs form highly stable RNPs and in contrast to SpyCas9, which cleaves a stoichiometric amount of DNA, SauCas9 is a multiple turnover enzyme. To our knowledge, this is the first report of such activity among Cas9 homologs. Furthermore, in contrast to SpyCas9, SauCas9 did not have any detectable additional nuclease activity on cleaved DNA products, yielding homogeneous products. Our findings illuminate distinct differences between two Cas9 homologs—both of which are widely used in various biotechnological and therapeutic applications—and add important insights for future development of CRISPR/Cas9 technologies.

## RESULTS

### *S. pyogenes* Cas9 binds sgRNA with a higher affinity than SauCas9 and both form active, sgRNA-dependent complexes with comparable *K*_1/2_ for sgRNA

CRISPR RNA (crRNA) and trans-activating crRNA (tracrRNA) together program Cas9-catalyzed endonucleolytic cleavage of DNA. The two RNAs can be bridged by a GAAA tetraloop, forming a single guide RNA (sgRNA) (Jinek et al. 2012), thus reducing the number of components and facilitating DNA targeting applications. The 3′-end harbors three stem loops in the *S. pyogenes* and two in the *S. aureus* sgRNAs (Nishimasu et al. 2014; Ran et al. 2015), which are recognized by the protein and are critical for forming an active Cas9-sgRNA RNP. Changing the 5′-proximal ∼20-nt sequence is enough to direct Cas9 to a specific site in the substrate DNA. However, not all DNA targets are cleaved with equal efficiency and specificity (Doench et al. 2014; Bisaria et al. 2017). sgRNA stem loops are critical for recognition and binding Cas9 (Briner et al. 2014; Mekler et al. 2016) and it is thought that one contributing reason for differences in cleavage efficiencies is the degree of 5′-end base-pairing with the 3′-region of the sgRNA (Thyme et al. 2016; Xu et al. 2017), causing a disruption of the stem loops recognized by Cas9.

**FIGURE 1.**
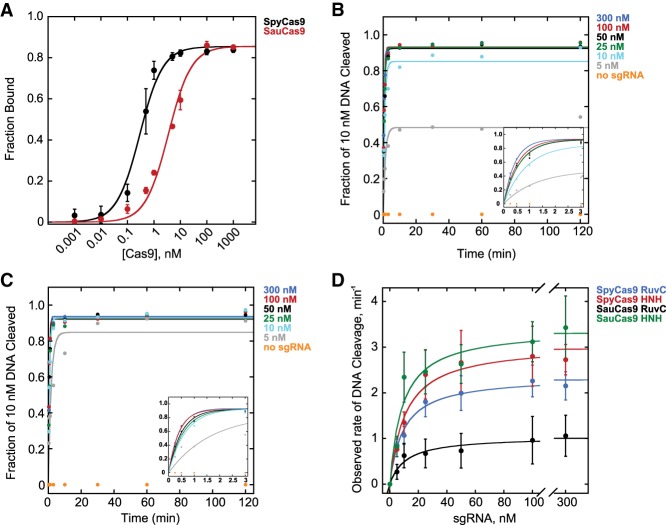
*S. pyogenes* and *S. aureus* Cas9 tightly bind their respective sgRNAs and form active RNPs. (*A*) Fraction bound of 50 pM 5′-^32^P-labeled sgRNA versus concentration of unlabeled SpyCas9 (black) or SauCas9 (red), resolved on a 6% native TBE acrylamide gel in the cold room. The *K*_*D*_ values for SpyCas9 and SauCas9 were 0.29 ± 0.08 nM (*R*^2^ = 0.99) and 3.4 ± 0.2 nM (*R*^2^ = 0.99), respectively. (*B–C*) Representative plots showing observed rates (*k*_obs_) of HNH domain-catalyzed hydrolysis of 10 nM 110mer DNA by 25 nM (*B*) SpyCas9 and (*C*) SauCas9 in the presence of 300 nM (blue), 100 nM (red), 50 nM (black), 25 nM (green), 10 nM (cyan), 5 nM (gray), or no sgRNA (orange). *Insets* feature early time points of the respective plots for clarity. (*D*) Dependence of *k*_obs_ on sgRNA concentration for SpyCas9 and SauCas9. (Blue) SpyCas9 RuvC domain-catalyzed cleavage: *k*_max_ = 2.3 ± 0.4 min^−1^, *K*_1/2_ = 8 ± 1 nM (*R*^2^ = 0.99); (red) SpyCas9 HNH domain-catalyzed cleavage: *k*_max_ = 3.0 ± 0.6 min^−1^, *K*_1/2_ = 9 ± 1 nM (*R*^2^ = 0.97); (black) SauCas9 RuvC domain-catalyzed cleavage: *k*_max_ = 1.0 ± 0.5 min^−1^, *K*_1/2_ = 10 ± 1 nM (*R*^2^ = 0.95); (green) SauCas9 HNH domain-catalyzed cleavage: *k*_max_ = 3.3 ± 0.5 min^−1^, *K*_1/2_ = 8 ± 2 nM (*R*^2^ = 0.94). All of the values are the result of at least three independent experiments and are reported as mean ± average deviation.

We designed SpyCas9 and SauCas9 sgRNAs (Table 1) to have a 20-nt-long targeting sequence (Jinek et al. 2012; Ran et al. 2015) while minimizing the degree of base-pairing between the 5′- and 3′-ends—approximated by quick structure prediction algorithms such as mfold (Zuker 2003)—and measured the binding affinity (*K*_D_) for the respective Cas9 homologs (Fig. 1A). sgRNAs were transcribed in vitro with T7 RNA Polymerase, purified on a denaturing acrylamide gel, and labeled with 5′-^32^P. Cas9 protein was titrated in the presence of 50 pM ^32^P-labeled sgRNA in 1× NEBuffer 3.1. RNA bound to Cas9 was resolved from the faster-migrating free sgRNA on a native acrylamide gel. The data were fit with a hyperbolic binding equation as described previously (Pollard 2010) (Materials and Methods). The *K*_D_ for SpyCas9 was 290 ± 80 pM, which is higher than previously reported values (∼10 pM) (Wright et al. 2015), possibly due to differences in buffer conditions and/or experimental approach. Given the high affinity of this complex, it is possible that the value is an underestimate of the strength of the interaction; however, we can definitively conclude that the SpyCas9 and the sgRNA used in this study form an RNP with a *K*_D_ at least in the pM range in NEBuffer 3.1. In contrast to SpyCas9, SauCas9 bound to its respective sgRNA with a weaker affinity of 3.4 ± 0.6 nM.

**TABLE 1.**
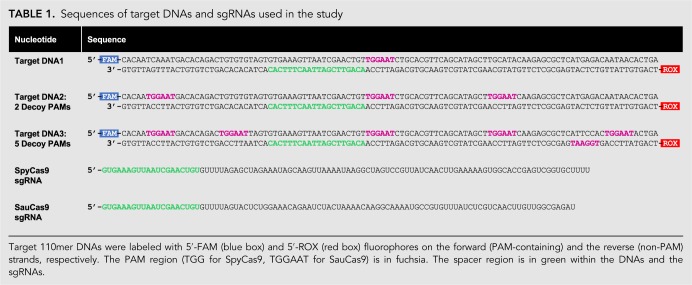
Sequences of target DNAs and sgRNAs used in the study

Having established that Spy- and SauCas9 homologs bind their respective sgRNAs, we measured the dependence of the rate and extent of DNA cleavage on the sgRNA concentration. sgRNAs were refolded by heating to 65°C and cooling to 4°C at 0.1°C/sec in a thermocycler. 25 nM Spy- or SauCas9 was preincubated in the presence of a variable concentration of sgRNA, ranging from 0 to 300 nM, for 15 min and reactions were initiated by addition of 10 nM 110mer double-stranded DNA1 (Table 1) harboring a 20-nt target sequence that was a perfect complement to the sgRNA as well as a TGGAAT PAM, which fulfills both Spy (NGG) and Sau (NNGRRT) PAM requirements (Mojica et al. 2009; Friedland et al. 2015). The DNA did not have any other occurrences of either the target sequence or the PAMs (Table 1, DNA1) and was labeled with a 5′-FAM fluorophore on the PAM-containing strand (cleaved by the RuvC domain) and a 5′-ROX fluorophore on the non-PAM strand (cleaved by the HNH domain). Reaction aliquots were quenched with a final concentration of 50 mM EDTA, 1% SDS, and 0.1 units/µL of Proteinase K and analyzed by capillary electrophoresis (CE) (Greenough et al. 2016). The fraction of DNA cleaved was plotted versus time, and fit with a single exponential equation describing the observed rate (*k*_obs_) and extent of DNA cleavage in the presence of a particular concentration of sgRNA (Fig. 1B,C) (Materials and Methods). Both Spy- and SauCas9 achieved ≥93% extent of cleavage of the DNA when the concentration of the sgRNA was at least stoichiometric to the 110mer target DNA. In contrast, <2% cleavage was observed in the absence of sgRNA (Fig. 1B,C). *k*_obs_ were plotted against the concentration of sgRNA (Fig. 1D) and fit with a hyperbolic equation (Materials and Methods), giving the maximal rate of cleavage (*k*_max_), when the reaction is not limited by sgRNA concentration, and the concentration of sgRNA required to achieve the half-maximal rate of DNA cleavage (*K*_1/2_). The *k*_max_ for the cleavage of the non-PAM DNA strand by the HNH domain was similar for SpyCas9 and SauCas9 (3.0 ± 0.6 min^−1^ and 3.3 ± 0.5 min^−1^, respectively). The *k*_max_ for the cleavage of the PAM-containing strand by the RuvC domain was modestly slower for SpyCas9 (2.3 ± 0.4 min^−1^) consistent with previous reports (Gong et al. 2018; Raper et al. 2018), and approximately threefold slower for SauCas9 (1.0 ± 0.5 min^−1^). The *K*_1/2_ values for both Spy- and SauCas9—measured for either PAM-containing or non-PAM strand cleavage—were between 8 nM and 10 nM (Fig. 1D), consistent with an efficient interaction between the Cas9 protein and sgRNA (Fig. 1A). It did not escape our attention that SpyCas9 achieved ∼50% reaction extent (∼5 nM DNA cleaved) in the presence of substoichiometric (5 nM) sgRNA, while SauCas9 achieved ∼95% reaction extent under the same conditions (Fig. 1B,C).

### *S. aureus* but not *S. pyogenes* Cas9 is a multiple turnover enzyme in vitro

**FIGURE 2.**
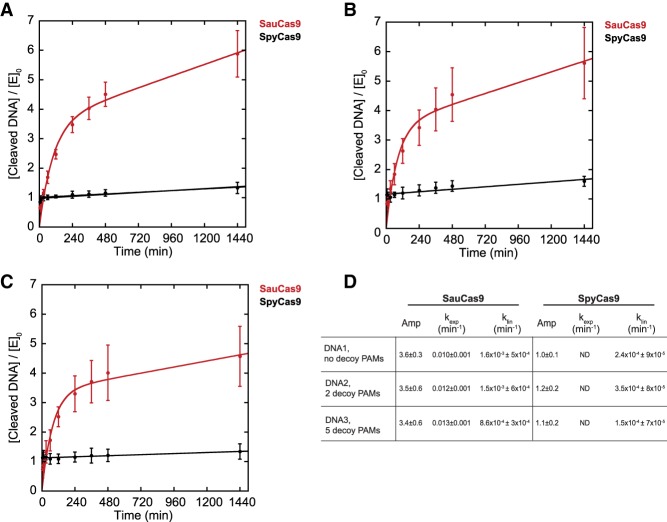
*S. aureus* Cas9 is a multiple turnover enzyme. A total of 25 nM SpyCas9 (black) or SauCas9 (red) was preincubated with 100 nM sgRNA for 15 min prior to the addition of 250 nM DNA. The amount of HNH-cleaved non-PAM strand of the DNA, divided by the enzyme concentration, was plotted versus time. (*A*) DNA1, no decoy PAMs. SauCas9: amplitude = 3.6 ± 0.3; *k*_exp_ = 0.010 ± 0.001 min^−1^; *k*_lin_ = 1.6 × 10^−3^ ± 6 × 10^−4^ min^−1^ (*R*^2^ = 0.98). SpyCas9: amplitude = 1.0 ± 0.1; *k*_lin_ = 2.4 × 10^−4^ ± 9 × 10^−5^ min^−1^ (*R*^2^ = 0.95). (*B*) DNA2, 2 decoy PAMs. SauCas9: amplitude = 3.5 ± 0.60; *k*_exp_ = 0.012 ± 0.001 min^−1^; *k*_lin_ = 1.5 × 10^−3^ ± 6 × 10^−4^ min^−1^ (*R*^2^ = 0.96). SpyCas9: amplitude = 1.2 ± 0.2; *k*_lin_ = 3.5 × 10^−4^ ± 8 × 10^−5^ min^−1^ (*R*^2^ = 0.83). (*C*) DNA3, 5 decoy PAMs. SauCas9: amplitude = 3.4 ± 0.6; *k*_exp_ = 0.013 ± 0.001 min^−1^; *k*_lin_ = 8.6 × 10^−4^ ± 3 × 10^−4^ min^−1^ (*R*^2^ = 0.96). SpyCas9: amplitude = 1.1 ± 0.2; *k*_lin_ = 1.5 × 10^−4^ ± 7 × 10^−5^ min^−1^ (*R*^2^ = 0.83). (*D*) Summary of the data in *A–C* for convenience. ND, the rate of the burst could not be resolved by manual quenching. All of the values are the result of at least three independent experiments and are reported as mean ± average deviation.

A prominent feature of SpyCas9 is an extremely slow rate of turnover. Recent work in vitro and in vivo (Sternberg et al. 2014; Jones et al. 2017; Gong et al. 2018; Raper et al. 2018) demonstrated that the Cas9•sgRNA RNP rapidly finds and cleaves the target DNA sequence but does not dissociate from the cleaved DNA. In contrast, another study involving SpyCas9 suggests that cleaved DNA strands may be released from the post-cleavage complex (Richardson et al. 2016); however, Cas9 is widely accepted to be a single turnover enzyme expected to cleave substrate DNA in approximately 1:1 stoichiometry with active RNP complexes. We performed experiments designed to replicate the previous observations of others by preincubating 25 nM Spy- or SauCas9 and 100 nM of respective sgRNA for 15 min and then added a 10-fold excess of 110mer DNA1 (250 nM) (Table 1), quenched reaction aliquots over a course of 24 h, and analyzed the reactions by CE as described above. As expected, within less than 5 min of initiating the reaction containing SpyCas9 there was ∼25 nM of cleaved product, which increased to 33 ± 5 nM over 24 h, suggesting that SpyCas9 is nearly 100% active in the presence of saturating sgRNA but there is a very low degree of turnover (Fig. 2A). Strikingly, after 24 h, SauCas9 resulted in 150 ± 20 nM cleaved DNA, suggesting that the enzyme turns over significantly faster than the *S. pyogenes* homolog. For both SpyCas9 and SauCas9, the reaction was described well by a single exponential followed by a linear (i.e., steady state) phase equation (Materials and Methods). The burst kinetics for SpyCas9 were too rapid to be resolved with manual quenching; however, the estimated burst amplitude was consistent with the SpyCas9 concentration of 25 nM (Fig. 2A). It is possible that initiating reactions with 10 mM Mg^2+^ (Gong et al. 2018; Raper et al. 2018) would result in an even faster burst, however, our experimental approaches would not be able to resolve such kinetic differences. It is unlikely that the equation fit to the SauCas9 data recapitulates a true burst because the amplitude is nearly fourfold higher than the SauCas9 concentration in the reaction (Fig. 2A). Interestingly, the linear phase of the reaction for SauCas9 was 1.6 × 10^−3^ ± 5 × 10^−4^ min^−1^, which is sevenfold faster than measured for SpyCas9 (Fig. 2A,D). Preincubating SpyCas9 or SauCas9 RNPs for 24 h at reaction conditions prior to addition of substrate DNA1 resulted in nearly identical results, strongly suggesting that the RNP is stable and retains full activity over the course of a 24-h reaction (data not shown), thus eliminating the possibility that the change in the rate of product formation is due to loss of active RNPs. Nuclease contamination in the protein stocks is also unlikely due to lack of DNA cleavage in the absence of sgRNA (Fig. 1B,C). Reported affinities of SpyCas9•sgRNA RNP for a target DNA are in the low nM range (Sternberg et al. 2014), so it is also unlikely that the pool of available substrate 110mer DNA1 approaches or decreases below the *K*_m_ over the period of the time course. Based on these assumptions, we next compared SpyCas9 and SauCas9 DNA cleavage activities with modified versions of 110mer DNA1 in order to better understand the differences between the two homologs.

### Presence of “decoy” PAMs does not significantly affect *S. pyogenes* Cas9 or *S. aureus* Cas9 activity

The 110mer DNA1 substrate in our study (Table 1, DNA1) contained one instance of the PAM, adjacent to the target sequence, which is not the case in genomic DNA. A genome is likely to contain numerous PAMs that occur, by chance, distal to the target sequence. Therefore, we tested how additional “decoy” TGGAAT PAMs affect the turnover activity of Spy- and SauCas9. We mutated the 110mer DNA1 substrate to contain either 2 or 5 decoy PAMs in addition to the “true” PAM located 3′- to the target sequence (Table 1). The target sequence within the DNA was not changed to avoid potential differences in editing efficiency observed with different targets and requisite sgRNAs (Doench et al. 2014; Thyme et al. 2016). Addition of extra PAMs resulted in a small decrease in the total DNA cleaved by SauCas9, from 150 ± 20 nM in the absence of decoy PAMs to 110 ± 30 nM in the presence of 5 decoy PAMs (Fig. 2A–C). The degree of cleavage observed with SpyCas9 was not significantly affected by presence of decoy PAMs. The measurable rates of reactions were not affected significantly for either homolog (Fig. 2B–D).

### *S. aureus* Cas9 releases cleaved product DNA faster than *S. pyogenes* Cas9

**FIGURE 3.**
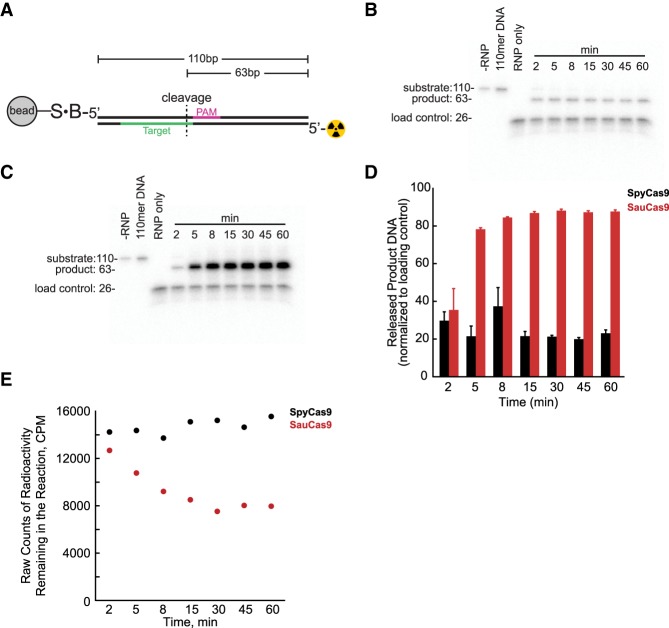
*S. aureus* Cas9 releases cleaved product DNA faster than *S. pyogenes* Cas9. (*A*) Cartoon illustrating the DNA1 substrate immobilized on a streptavidin magnetic bead, used for monitoring release of cleaved products in solution. S, streptavidin; B, 5′-biotin; radioactivity symbol, 5′-^32^P. The PAM-containing strand possessed a 5′-biotin and the non-PAM strand was labeled with a 5′-^32^P. DNA strands are color-coded the same as in Table 1: The PAM region (5′-TGGAAT) on the strand containing a 5′-biotin is in fuchsia and the target sequence (5′-ACAGTTCGATTAACTTTCAC) complementary to the sgRNAs, on the strand containing a 5′-^32^P, is in green. (*B*,*C*) Release of cleaved DNA product 63mer, over time, by (*B*) SpyCas9 and (*C*) SauCas9 resolved on a 15% TBE urea denaturing gel. (*D*) Quantification of released product in *B*,*C* normalized to a 26mer single-stranded DNA control oligo; (black bars) SpyCas9 RNP, (red bars) SauCas9 RNP. Data are the result of two experimental replicates and reported as mean ± average deviation. (*E*) Total radioactive signal remaining in the reaction from *D*, measured by liquid scintillation, with SpyCas9 (black circles) and SauCas9 (red circles).

A possible explanation for the differences in the rate of turnover between SpyCas9 and SauCas9 may be the rate of product release. We designed an assay to monitor the appearance of released cleaved DNA product in solution (Materials and Methods). Briefly, instead of 5′-FAM and 5′-ROX fluorophores, DNA1 was made to contain a 5′-biotin and a 5′-^32^P on PAM-containing (“forward”) and non-PAM (“reverse”) strands, respectively (Fig. 3A). The DNA was immobilized on a magnetic streptavidin bead and incubated in the presence of excess Spy- or SauCas9 RNP. Appearance of 63mer cleaved DNA in solution was monitored over time and resolved on a Tris-Borate-EDTA-Urea gel (Fig. 3B,C). Released product was normalized to a nonspecific control oligo included in the Cas9 RNP premixes (Fig. 3D). After 15 min, the SauCas9 had more cleaved DNA released into solution than SpyCas9 by fourfold (Fig. 3D), suggesting that one possible explanation for the difference in the rate of turnover is that SauCas9 may have a faster rate of product release, than SpyCas9. Measurement of the total amount of radioactivity left over in the reaction (i.e., immobilized on the beads) by liquid scintillation, resulted in a comparable amount of signal for all timepoints with SpyCas9 while there was a decrease in the amount of radioactivity in the reactions with SauCas9, further corroborating that the cleaved DNA was released from the complex.

In spite of appearance of cleaved DNA in solution, this assay cannot resolve if SauCas9 was still attached to any of the strands of the DNA or RNA; however, the ability of the enzyme to cleave an excess of substrate DNA (Fig. 2) is consistent with the model that upon release of the product, SauCas9 is able to perform another round of catalysis.

### *S. aureus* Cas9 does not have detectable post-cleavage trimming activity

**FIGURE 4.**
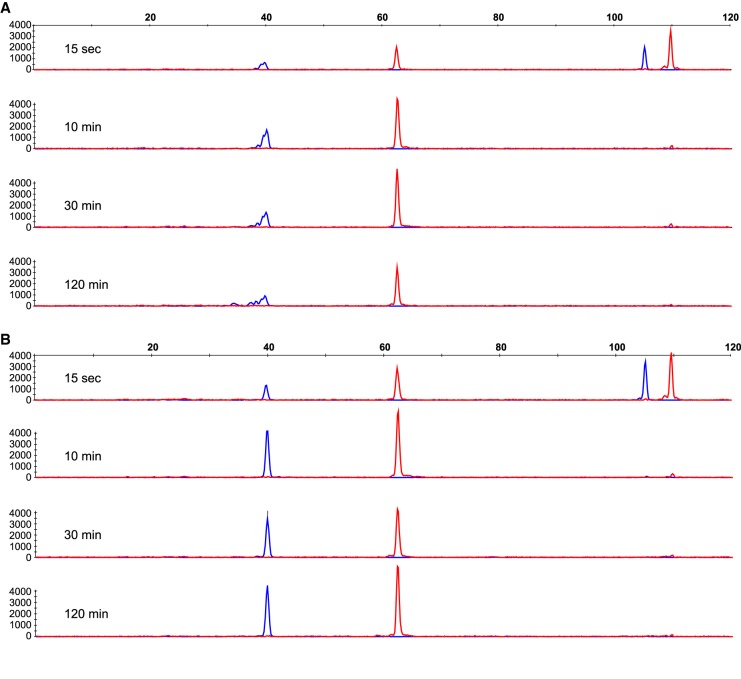
*S. aureus* Cas9 does not exhibit detectable post-cleavage trimming activity on cleaved DNA. (*A*,*B*) Representative capillary electrophoresis of 110mer DNA labeled with 5′-FAM (blue trace) on the PAM-containing strand and 5′-ROX (red trace) on the non-PAM strand hydrolyzed by (*A*) SpyCas9 or (*B*) SauCas9 at reaction time points of 15 sec, 10 min, 30 min, and 120 min. Data are plotted as relative fluorescence versus DNA oligomer length. The trend was consistently observed over the course of our experimentation (*n* > 10) with SpyCas9 and SauCas9.

Lastly, we were curious about the nature of the cleaved DNA products. Recent work demonstrated that SpyCas9 exhibits RuvC-catalyzed post-cleavage exonuclease activity on cleaved DNA products (Jinek et al. 2012; Ma et al. 2015; Stephenson et al. 2018). Reactions were carried out at single turnover conditions, described above, with Spy- or SauCas9 and DNA1 cleavage products were resolved by CE (Table 1; Fig. 4). The products of reactions containing SpyCas9 were consistent with previous observations: The non-PAM strand, labeled with a 5′-ROX fluorophore, resulted in a single homogenous peak, while the PAM-containing strand—labeled with a 5′-FAM fluorophore—resulted in a series of smaller peaks, indicating that the PAM-distal fragment of the PAM-containing strand was cut in various locations, degraded further upon cleavage or both (Fig. 4A). No significant degradation of cleaved DNA products was observed in the reaction containing SauCas9 (Fig. 4B). Both the PAM-containing (5′-FAM, cleaved by RuvC) and the non-PAM (5′-ROX, cleaved by HNH) strands yielded single homogenous peaks that increased in magnitude over the course of the reaction (Fig. 4B). Because the reaction substrates were labeled on the 5′-ends (Table 1), we cannot rule out degradation of the PAM-proximal PAM-containing strand nor the PAM-distal fragment of the non-PAM strand; however, these data provide an insight into another important mechanistic difference between the *S. pyogenes* and *S. aureus* Cas9 homologs that is to be considered in applications of the CRISPR/Cas9 technology.

## DISCUSSION

SpyCas9 is the best-characterized Cas9 enzyme due in large part to its widespread adoption as a genome editing tool. SauCas9 shares 17% sequence identity with SpyCas9 and has been less intensively reported on. Our in vitro data suggest that SauCas9 is a multiple-turnover enzyme while SpyCas9 is not. This finding may provide some insight into previous observations suggesting that SauCas9 is modestly more active than SpyCas9 in cells (Xie et al. 2018). Previous reports and published protocols show editing by transfecting HEK293 cells with 100 pmol (100 µM) of preformed sgRNA-Cas9 RNP (Lin et al. 2014; DeWitt et al. 2017; Lingeman et al. 2017) and it would be of great interest to directly compare the activity of SpyCas9 and SauCas9, delivered as RNPs, in vivo. DNA cleavage in vitro may not completely correlate with editing in vivo; however, the fact that SauCas9, but not SpyCas9, is able to undergo multiple rounds of catalysis, suggests a fundamental mechanistic difference between the homologs. To our knowledge, this is the first report of a multiple-turnover Cas9. The rate of turnover is slow but it is significantly faster than for SpyCas9. Indeed, it is possible that if a double-stranded DNA break were to be repaired by cellular machinery to its original state (i.e., without any resulting indels), a faster rate of turnover by SauCas9 should result in more attempts to re-introduce the double-stranded DNA break at the site of interest and thus might result in a higher degree of editing.

It is somewhat surprising that in spite of faster turnover and a longer (i.e., less common) PAM, the amount of product formation was modestly decreased in DNA2 and DNA3—containing additional PAMs—for SauCas9 but not SpyCas9. One possible model, which will be the subject of future studies, is that while SauCas9 may have a faster rate of product release, it might bind a substrate PAM sequence with a higher affinity than SpyCas9, irrespective of the adjacent target sequence.

Mechanistic work demonstrates that SpyCas9 cleaves the PAM-containing strand of the DNA in variable locations and that cleaved DNA is modestly degraded post-cleavage (Jinek et al. 2012; Ma et al. 2015; Stephenson et al. 2018), while our data show that SauCas9 cleaves in a single location and no detectable degradation products accumulate. Previous data (Jinek et al. 2014; Richardson et al. 2016; Stephenson et al. 2018) suggest that the PAM-containing strand of the DNA may be more flexible in the SpyCas9•sgRNA•target DNA complex, which might contribute to heterogeneity in the cleavage sties. In our experimental setup, CE provides single nucleotide resolution and the presence of uniform peaks for SauCas9-catalyzed reactions strongly suggests that the DNA is cleaved in a single location. It is possible that the PAM-containing strand is more rigid in the SauCas9•sgRNA•target DNA complex than in the analogous complex with SpyCas9. An alternative model consistent with our data is that SauCas9 exhibits a faster rate of product release and thus does not have sufficient time to degrade the DNA post-cleavage.

In this study, we compared DNA cleavage activity of *S. pyogenes* and *S. aureus* Cas9 in the presence of saturating sgRNA, in vitro. Our data provide novel insights into the mechanism of catalysis for these enzymes. SauCas9 is smaller by more than 300 amino acids, greatly reducing the challenges of vector-based delivery into cells (Senis et al. 2014; Ran et al. 2015), is a multiple-turnover enzyme, and cleaves DNA in a single location without further degradation. Taken together, these findings suggest that SauCas9 may be an attractive alternative or complement to SpyCas9 and could possibly be leveraged for future biotechnological and therapeutic applications.

## MATERIALS AND METHODS

### Reagents

*S. pyogenes* Cas9 (# M0386M), EnGen sgRNA Synthesis Kit, *S. pyogenes* (# E3322S), HiScribe T7 High Yield RNA Synthesis Kit (E2040S), 2× RNA Loading Dye (# B0363S), Nucleoside Digestion Mix (M0649S), Q5 Hot Start High-Fidelity 2× Master Mix (# M0494L), Monarch PCR & DNA Cleanup Kit (# T1030L), Proteinase K (# P8107S), Shrimp Alkaline Phosphatase (# M0371S), T4 Polynucleotide Kinase (# M0201S), Streptavidin Magnetic Beads (# S1420S), and NEBuffer 3.1 (# B7203S) with a 1× composition of 100 mM NaCl, 50 mM Tris-HCl, 10 mM MgCl_2_, 100 μg/ml BSA, pH 7.9 at 25°C were all from New England Biolabs. Both *S. pyogenes* and *S. aureus* Cas9 were purified at New England Biolabs using standard liquid chromatography protein purification techniques. Protein stock concentration for both Spy- and SauCas9 was measured by absorbance of 280 nm light on a NanoDrop instrument (A_280_) as well as Bio-Rad Bradford assays per manufacturer protocol. All DNA oligomers were ordered from Integrated DNA Technologies. The Zymo RNA Clean & Concentrator-5 kit (#R1016) was purchased from Zymo Research. The SequaGel–UreaGel (# EC-833) system was from National Diagnostics. Yeast tRNA (# AM7119), 6% (# EC62652), and 15% (# EC6885) Tris-Borate EDTA gels were from Invitrogen/Thermo Fisher Scientific. γ-32P-ATP was from PerkinElmer (# BLU002A100UC).

### sgRNA transcription, purification, and labeling

*S. aureus* sgRNA was transcribed using the single-stranded DNA template 5′-ATCTCGCCAACAAGTTGACGAGATAAACACGGCATTTTGCCTTGTTTTAGTAGATTCTGTTTCCAGAGTACTAAAACACAGTTCGATTAACTTTCACTATAGTGAGTCGTATTAATTTCGA and an oligo that is complementary to the T7 promoter region 5′-TCGAAATTAATACGACTCACTATAG. Oligos were transcribed using the NEB HiScribe T7 High Yield RNA Synthesis Kit according to manufacturer protocol. *S. pyogenes* sgRNA was transcribed with the EnGen sgRNA Synthesis Kit according to manufacturer protocol with the following oligo added to the reaction: 5′-TTCTAATACGACTCACTATAGTGAAAGTTAATCGAACTGTGTTTTAGAGCTAGA. Transcription products were purified as described previously (Linpinsel and Conn 2012) with small modifications: Reactions were quenched with an equal volume of 2× NEB RNA Loading Dye and resolved on a 10% SequaGel (10% acrylamide, 7.5 M urea) in 1× TBE buffer. RNA bands were visualized by UV shadowing, cut out, crushed with a micro spatula, and soaked overnight at 4°C in 300 mM NaOAc, pH 5.5. Eluted RNA was filtered with 0.22 µm pore syringe-driven PVDF filter, precipitated with 3 vol of 95% ethanol at −20°C for 16 h, and resuspended in water. sgRNA concentration was determined with the NanoDrop by monitoring absorbance of 260 nm light (OD_260_) at room temperature and converted to molar concentration with the NEBioCalculator (https://nebiocalculator.neb.com/#!/ssrnaamt), which uses the formula:

moles ssRNA(mol)=mass of ssRNA(g)/((length of ssRNA (nt)×321.47g/mol)+18.02g/mol).

Determination of extinction coefficients by enzymatic digest of the sgRNAs (Cavaluzzi and Borer 2004; Room 2014) with the NEB Digestion Mix or with in silico methods (Integrated DNA Technologies OligoAnalyzer Tool) (Warshaw and Tinoco 1966; Cantor et al. 1970; Cavaluzzi and Borer 2004) yielded sgRNA concentrations for Spy- and SauCas9 orthologs that were both modestly but equally higher (∼1.3-fold) and comparable to the values determined by measuring OD_260_ with the NanoDrop.

In vitro-transcribed and gel-purified sgRNAs were incubated with Shrimp Alkaline Phosphatase, per manufacturer protocol, and purified with the Zymo RNA Clean & Concentrator-5 kit. Subsequently, the sgRNAs were labeled with a 5′-^32^P using T4 Polynucleotide Kinase according to manufacturer protocol and purified with the Zymo RNA Clean & Concentrator-5 kit.

### sgRNA binding measured by gel shift

sgRNA binding to Cas9 proteins was measured with an electrophoretic mobility shift assay (EMSA). Unlabeled Cas9 was titrated in the presence of 50 pM 5′-^32^P-sgRNA in NEBuffer 3.1. Reactions were incubated for 40 min at 22°C, separated on a 6% native TBE gel in the cold room, visualized by phosphorimaging, and quantified with ImageQuant software. The fraction of sgRNA bound was plotted versus Cas9 concentration and fit using KaleidaGraph software with a hyperbolic equation as described previously (Pollard 2010; Wright et al. 2015):

$$\mathrm{Fraction}\;\mathrm{bound}=\frac{E}{K_{D}+E},$$

where *E* is the concentration of Cas9 in the reaction.

### DNA amplification and purification

Forward and reverse DNA primers labeled with a 5′-FAM and a 5′-ROX fluorophore, respectively, were used to amplify single-stranded unlabeled DNA with the target sequence and a PAM (Supplement 1)—using the NEB Q5 Hot Start High-Fidelity 2× Master Mix per manufacturer protocol—thus creating various 110mers used in the study (Table 1). PCR reactions were purified with the Monarch PCR & DNA Cleanup Kit per manufacturer protocol. DNA1 containing a 5′-biotin on the PAM-containing strand and a 5′-^32^P on the non-PAM strand was created in exactly the same way, except the forward and reverse PCR primers contained a 5′-biotin and a 5′-^32^P, respectively. The forward primer was synthesized by IDT and the reverse primer was labeled with a 5′-^32^P using T4 PNK and γ-^32^P-ATP according to manufacturer protocol. T4 PNK and excess γ-^32^P-ATP were removed with the Monarch PCR & DNA Cleanup Kit.

### DNA cleavage assays

All reactions were performed in NEBuffer 3.1 (see “Reagents” above for composition) and carried out at 22°C.

Single turnover experiments to determine the *k*_max_ and *K*_1/2_ for sgRNA: 25 nM Cas9 was preincubated with different concentrations of sgRNAs for 15 min and reactions were initiated by addition of 110mer DNA (10 nM final concentration). Reaction time points were acquired by quenching 2 µL aliquots by combining with 2 µL of 2× quench mix containing 100 mM EDTA, 2% SDS, and 0.2 units/µL of Proteinase K. The quench mix was used not more than 10 min after addition of Proteinase K to insure maximum proteolytic activity. Quenched reactions were resolved on the Applied Biosystems 3730xl instrument and analyzed using the PeakScanner software (Applied Biosystems) (Greenough et al. 2016). The fraction of DNA cleaved was calculated by dividing the integrated peak area of the product peak by the combined integrated area of the product and the substrate peaks. The fraction of DNA cleaved was plotted versus time and fit with a single exponential equation:

$$\mathrm{Fraction}\;\mathrm{of}\;\mathrm{DNA}\;\mathrm{cleaved}=A\times (1-e^{-kt}),$$

where *t* is time, *A* is amplitude, and *k* is the observed rate constant, *k*_obs_.

Observed rate constants were plotted versus sgRNA concentration and fit to a hyperbolic equation:

$$\mathrm{Observed}\;\mathrm{rate}=\frac{k_{\max}\times R}{K_{1/2}+R},$$

where the *k*_max_ is the maximal rate of the reaction when not limited by sgRNA concentration, *R* is the concentration of sgRNA in the reaction, and *K*_1/2_ is the concentration of sgRNA required to achieve half-maximal rate of DNA cleavage.

Multiple turnover reactions were performed exactly as described above except the concentration of the DNA was 250 nM. It is possible that initiating reactions with 10 mM Mg^2+^ (Gong et al. 2018; Raper et al. 2018) would result in a faster burst; however, our experimental approaches would not be able to resolve such kinetic differences. The fraction of DNA product cleaved was multiplied by 250 nM total DNA in the reaction to obtain nM product × min^−1^ and divided by 25 nM enzyme concentration to obtain rates in units of min^−1^. The reactions were fit to:

$$\mathrm{Cleaved}\;\mathrm{DNA}=A\times (1-e^{-k_{\exp}t})+k_{\mathrm{lin}}t,$$

where *A* is amplitude, *t* is time, *k*_exp_ and *k*_lin_ are the exponential and linear rates describing DNA cleavage, respectively.

### Cleaved DNA product release in solution

Four hundred microliters of 20 nM 5′-biotinylated DNA1, labeled with ^32^P, was immobilized on 320 µg of streptavidin magnetic beads in binding buffer (20 mM Tris pH 7.5, 500 mM NaCl, 1 mM EDTA), per manufacturer protocol, for 30 min with gentle agitation at room temperature. The beads were then washed three times with binding buffer followed by three washes with 1× NEBuffer 3.1 reaction buffer supplemented with 100 ng/µL yeast tRNA. The beads were then resuspended in 80 µL of reaction buffer (1× NEB 3.1 with 100 ng/µL yeast tRNA). For each timepoint, 5 µL of beads with immobilized DNA1 was combined with 40 µL of 25 nM Cas9•sgRNA RNP in NEBuffer 3.1 supplemented with 100 ng/µL yeast tRNA and a nonspecific 5′-^32^P-labeled 26mer ssDNA control oligo to ensure that a comparable amount of RNP was added to each reaction. The beads were allowed to collect on the magnet for 2 min prior to the desired time point and a 20 µL aliquot of the supernatant was combined with 4 µL of 100 mM EDTA, followed by the addition of 20 µL of 95% formamide and 0.02% xylene cyanol solution, incubated at 95°C for 2 min, resolved on a 15% TBE, 7.5 M Urea gel in 1× TBE buffer, visualized with storage phosphor, and the bands were quantified with ImageQuant software.

The beads were resuspended in the remaining volume of the reaction (∼25 µL) and measured with liquid scintillation to compare the amount of radioactive signal remaining in each reaction.

## SUPPLEMENTAL MATERIAL

Supplemental material is available for this article.

## Supplementary Material

Supplemental Material
