# Supplementary material for: Staphylococcus aureus Cas9 is a multiple-turnover enzyme
Source: RNA. 2019 Jan;25(1):35–44. doi: 10.1261/rna.067355.118 (PMC6298560; doi:10.1261/rna.067355.118)
Supplement: Supplemental Material [file supp_25_1_35__index.html]

Staphylococcus aureus Cas9 is a multiple-turnover enzyme — Staphylococcus aureus Cas9 is a multiple-turnover enzyme — Supplemental Material 

# *Staphylococcus aureus* Cas9 is a multiple-turnover enzyme

## Supplemental Material

- Supplemental\_Data.xlsx
